# Anesthetic Management of a Cesarean Section in a Parturient With Von Hippel-Lindau Disease

**DOI:** 10.7759/cureus.96942

**Published:** 2025-11-16

**Authors:** Yuko Kato (Araki), Masahiko Bougaki

**Affiliations:** 1 Anesthesiology and Pain Relief Center, University of Tokyo Hospital, Tokyo, JPN; 2 Anesthesiology, The Institute of Medical Science, The University of Tokyo Hospital, Tokyo, JPN

**Keywords:** cesarean section, general anesthesia, mode of delivery, neuraxial anesthesia, von hippel-lindau disease

## Abstract

Von Hippel-Lindau (VHL) disease is a rare autosomal-dominant genetic disorder characterized by the development of multiple hemangioblastomas within the central nervous system. The anesthetic management of parturients with VHL is challenging, whether general or neuraxial anesthesia is used. Here, we report the case of a parturient with VHL whose cesarean section was successfully performed under general anesthesia. Anesthetic evaluation well in advance of delivery and the patient’s fully informed choice were important for successful management.

## Introduction

Von Hippel-Lindau (VHL) disease is a rare autosomal dominant genetic disorder characterized by the development of multiple hemangioblastomas within the cerebellum, spinal cord, and retina. VHL is also associated with visceral tumors, such as renal cell carcinoma, pancreatic cysts, and pheochromocytomas [1]. The estimated prevalence of VHL is approximately one in 36,000-50,000 live births, and the disease is associated with reduced life expectancy, with reported mean ages of death ranging from 40 to 60 years [2]. It remains unclear whether pregnancy affects the progression of VHL lesions. Given its potentially severe consequences, early diagnosis and rigorous surveillance are essential to minimize morbidity and mortality.

The anesthetic management of parturients with VHL is challenging for clinicians, whether general or neuraxial anesthesia is used, as they face the risk of hemangioblastoma rupture. The top priority of anesthetic management is to avoid procedures that may alter intracranial pressure and to minimize hemodynamic changes, which may be further complicated by the physiological changes associated with pregnancy.

Here, we report the case of a parturient with VHL whose cesarean section was successfully performed under general anesthesia.

## Case presentation

A 29-year-old nulliparous woman (height, 158 cm; pre-pregnancy weight, 48 kg) was referred to our obstetric anesthesia clinic at 31 weeks of pregnancy. She had been diagnosed with VHL two years prior. The diagnosis of VHL was largely incidental, as the patient had complained of only mild neck pain at the time, which resolved spontaneously within a few weeks.

**Figure 1:**
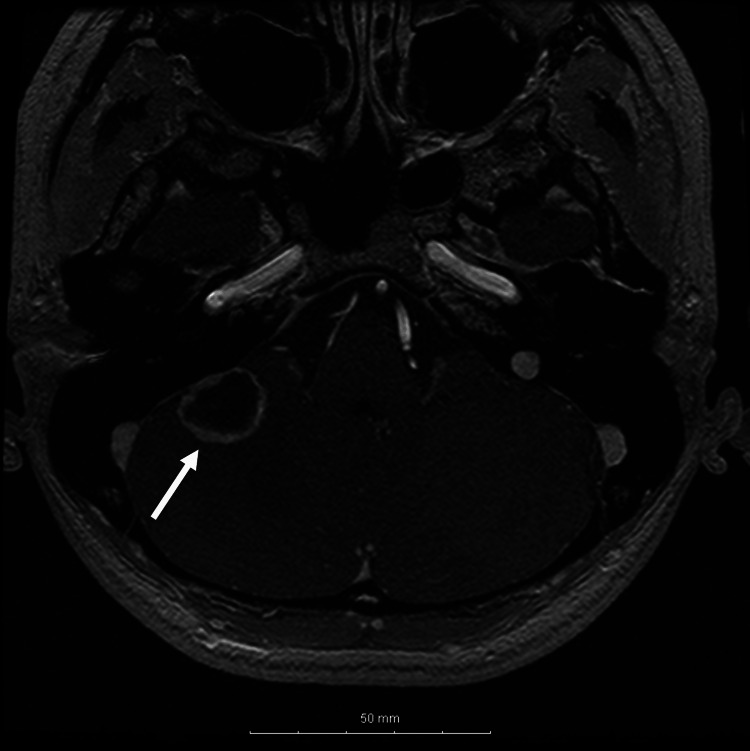
Magnetic resonance imaging of the brain performed six months before pregnancy A hemangioblastoma in the right cerebellum is visible on a T1-weighted image with gadolinium-based contrast.

**Figure 2:**
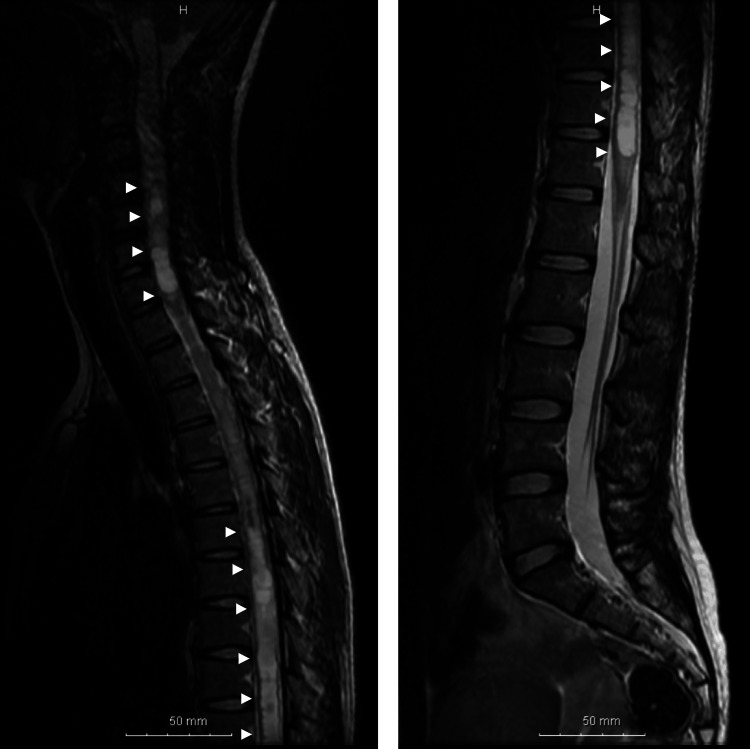
Magnetic resonance imaging of the spinal cord performed six months before pregnancy A T2-weighted image shows syringomyelia extending from the upper cervical region to T12.

Although she remained asymptomatic, magnetic resonance imaging performed six months before pregnancy revealed hemangioblastomas in the right cerebellum (Figure 1) and spinal cord (C4/5 and T7), accompanied by syringomyelia extending from C2 to T12 (Figure 2).

**Figure 3:**
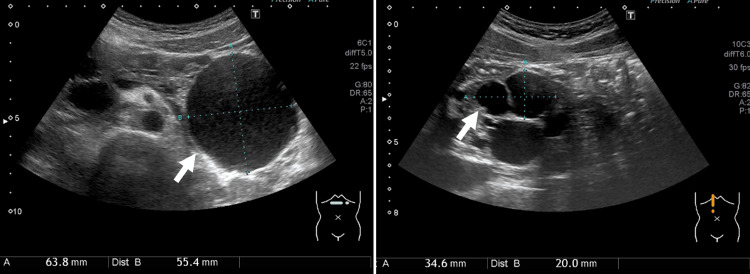
Abdominal ultrasonography performed two months before pregnancy Multiple cystic lesions are seen in the pancreas on abdominal ultrasonography.

**Figure 4:**
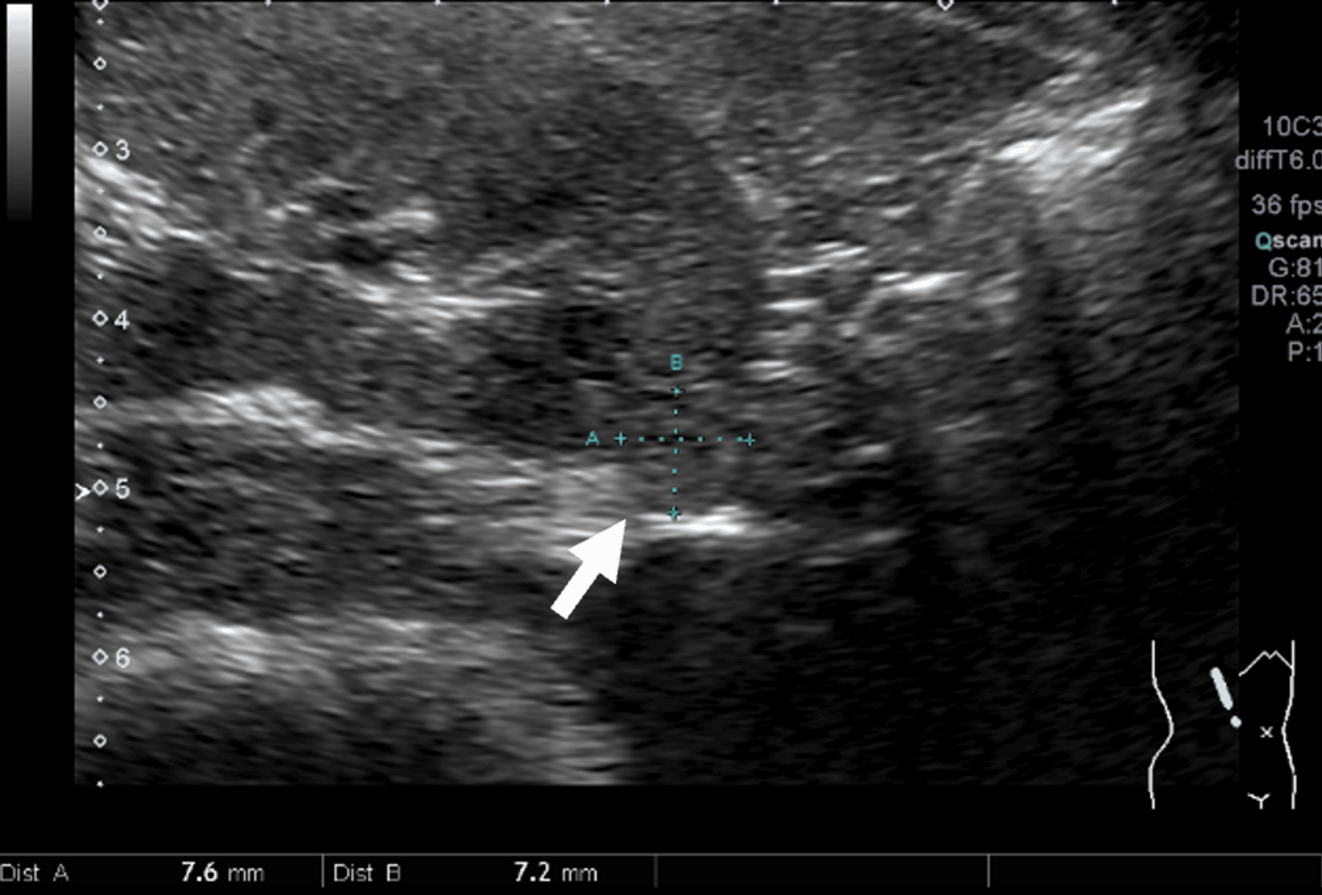
Abdominal ultrasonography performed four months before pregnancy A small renal mass, suspected to be renal cell carcinoma, is seen in the right kidney on abdominal ultrasonography.

Abdominal ultrasonography revealed multiple pancreatic cysts (Figure 3) and a small renal cell carcinoma in the right kidney (Figure 4).

Her blood pressure (BP) remained normal during pregnancy, and no clinical signs suggestive of pheochromocytoma were observed. Therefore, no additional screening tests were performed.

Upon examination, she had no neurological symptoms, and her prenatal course was unremarkable. The fetus was in the vertex position, but the mode of delivery had not yet been determined. After explaining to her and her husband the merits and risks of several anesthetic techniques, especially regarding the potential for hemangioblastoma rupture, we offered the following three options: (1) vaginal delivery with epidural labor analgesia; (2) cesarean section under epidural anesthesia (without spinal anesthesia); and (3) cesarean section under general anesthesia with tracheal intubation.

At her second visit to the obstetric anesthesia clinic at 36 weeks of pregnancy, she chose to undergo an elective cesarean section under general anesthesia, which was scheduled for 37 weeks of pregnancy.

However, the following day she returned to the hospital complaining of increased uterine contractions, and an emergency cesarean section was performed (Table 1). On arrival at the operating room, her BP was 117/56 mmHg, and her heart rate (HR) was 96 beats/min. After administering a moderate dose of remifentanil (0.3 µg/kg/min for 10 min) to minimize BP fluctuations associated with tracheal intubation, general anesthesia was induced with propofol 80 mg, fentanyl 100 µg, and rocuronium 60 mg. Bag-mask ventilation was minimized to prevent aspiration. The patient was intubated 2 min after induction, with a mild elevation in BP (134/93 mmHg; HR, 88 beats/min), and the operation was started immediately. The baby was delivered 3 min after skin incision, with Apgar scores of 7 at 1 min and 9 at 5 min. Anesthesia was maintained with a continuous infusion of propofol (4-5 mg/kg/h) and remifentanil (0.1-0.3 µg/kg/min). A total of 500 µg of fentanyl was administered throughout the operation. The patient remained hemodynamically stable throughout the operation and was extubated in the operating room under stable conditions (BP, 103/64 mmHg; HR, 85 beats/min). Postoperative analgesia consisted of fentanyl-based intravenous patient-controlled analgesia as well as periodic intravenous acetaminophen and oral loxoprofen. The patient’s postoperative course was unremarkable, and she and her baby were discharged on postoperative day 6.

**Table 1:** Procedural data

Procedure	Emergency cesarean section
Gestational age	36+4 weeks
Skin incision to delivery	3 min
Apgar scores (1 min/5 min)	7/9
Method of anesthesia	General anesthesia
Operation time	45 min
Anesthesia time	88 min
Estimated blood loss (including amniotic fluid)	790 mL

## Discussion

A previous case series of pregnancies associated with VHL showed that the majority of patients remained asymptomatic during pregnancy [3]. In contrast, maternal VHL-related complication rates have recently been reported to be as high as 17% during pregnancy, with frequent life-threatening complications for both the mother and fetus [4]. Whether pregnancy affects the progression of VHL lesions, particularly hemangioblastomas and pheochromocytomas, remains controversial. Clinicians should consider the possibility of pheochromocytomas when managing parturients with VHL. Although pheochromocytoma during pregnancy poses a serious risk to both the mother and fetus, outcomes are significantly better when the diagnosis is made early in the antenatal period compared to during labor or immediately postpartum [5].

Avoiding bleeding from hemangioblastomas is paramount in the anesthetic management of surgical patients with VHL. The hypertensive response caused by tracheal intubation or surgical stimuli should be minimized, typically using deep general anesthesia. However, in the setting of obstetric anesthesia, deep general anesthesia is not the preferred method, given the resulting effects on the fetus. Current literature lacks sufficient evidence to determine the ideal anesthetic management for parturients with VHL undergoing cesarean section.

Spinal anesthesia is the preferred technique for cesarean section. Although a case of successful cesarean section under spinal anesthesia in a patient with VHL has been reported [6], spinal anesthesia is usually considered a contraindication in patients with central nervous system (CNS) tumors because of the fear of bleeding from the tumor or cerebral herniation if associated with elevated intracranial pressure. Therefore, we considered it prudent to avoid spinal anesthesia in our patient.

Epidural anesthesia is generally considered safer than spinal anesthesia in patients with CNS tumors. Epidural administration does not directly alter intracranial pressure, whereas spinal anesthesia may cause intracranial pressure changes due to dural puncture. There have been several case reports on the successful use of epidural anesthesia in patients with VHL for labor analgesia [7,8] and cesarean section [8-13]. Although epidural anesthesia may carry the same risk as spinal anesthesia in the event of an inadvertent dural puncture, it remains a desirable option, particularly if vaginal delivery is considered, due to its ability to minimize BP fluctuations during labor and to accommodate possible physiological changes in the CNS during the second stage of labor [14]. Our patient was neurologically asymptomatic, and no lesions were detected in the lumbar spinal region. Taken together, we believe epidural anesthesia was a reasonable option for our patient, whether she chose vaginal delivery or cesarean section.

General anesthesia has been used for cesarean section in patients with VHL [14,15], especially when combined with other surgical procedures, such as pheochromocytoma resection [16], posterior fossa craniotomy for hemangioblastoma resection [17,18], or intracranial pressure monitor insertion [19]. General anesthesia is the only method of choice for certain life-threatening maternal or fetal situations. It may also be prudent to use general anesthesia if lumbar spinal lesions cannot be ruled out [15] or if increased intracranial pressure is suspected [18,19]. However, compared to neuraxial anesthesia, general anesthesia carries a higher risk of hypertensive responses during surgery, which may lead to bleeding from hemangioblastomas. Moreover, if the patient is clinically stable, the fetal depressant effects of general anesthesia must be carefully considered.

Considering these factors, the current best practice is as follows: the mode of anesthesia and delivery in parturients with VHL should be determined on a case-by-case basis based on an individual risk-to-benefit ratio [1]. As our patient had no definitive contraindications to either general or epidural anesthesia, her preferences played a key role in decision-making. It is also important to note that anesthesiologists played a critical role in both determining the mode of delivery and providing anesthesia. In our institution, anesthetic referral is made in most cases after obstetricians have decided on the delivery mode, which was not the case in our patient. On the first visit to the obstetric anesthesia clinic, we provided a thorough explanation of the merits and risks of several anesthetic techniques, including the choice of delivery mode. She and her family took time to consider the options and, by the second appointment, opted for a cesarean section under general anesthesia. As a result, she was well prepared, both mentally and physically, for the procedure, although she eventually underwent an emergency cesarean section.

## Conclusions

In summary, the management of parturients with VHL presents several anesthetic challenges. Anesthetic evaluation well in advance of the delivery and the patient’s fully informed choice are essential for successful peripartum management, including decisions regarding the mode of delivery and safe anesthetic care.
